# Permeable characteristics of surface film deposited on LiMn_2_O_4_ positive electrode revealed by redox-active indicator

**DOI:** 10.1186/s40580-021-00272-9

**Published:** 2021-07-14

**Authors:** Hyun-seung Kim, Jin Hyuk Yang, Ji Woo Han, Le Thi Thao, Ji Heon Ryu, Seung M. Oh, Ki Jae Kim

**Affiliations:** 1 Department of Chemical and Biological Engineering, Seoul National University, 1 Gwanak-ro, Gwanak-gu, Seoul, 08826 Republic of Korea; 2 Advanced Batteries Research Center, Korea Electronics Technology Institute, 25, Saenari-ro, Seongnam, 13509 Republic of Korea; 3 Department of Energy Engineering, Konkuk University, 120 Neungdong-ro, Gwangjin-gu, Seoul, 05029 Republic of Korea; 4 Graduate School of Knowledge-based Technology and Energy, Korea Polytechnic University, 237 Sangidaehak-ro, Siheung-si, Gyeonggi-do 15073 Republic of Korea

**Keywords:** Lithium-ion batteries, Electrolytes, Permeable characteristics, Surface film, Positive electrode

## Abstract

Herein, the ferrocene redox indicator-based surface film characteristics of spinel lithium manganese oxide (LMO) were evaluated. The pre-cycling of spinel LMO generated a film on the LMO surface. The surface film deposited on LMO surface suppresses further electrolyte decomposition, while the penetration of approximately 0.7 nm-sized redox indicator is not prevented. The facile self-discharge of LMO and regeneration current from the ferrocenium molecule was observed from the redox indicator in a specifically designed four-electrode cell. From this electrochemical behavior, a small-sized HF molecule attack on the LMO surface through a carbonate-based electrolyte-derived film is defined; hence, the prevention of small-sized molecules into the deposited surface film is crucial for the enhancement of LiMn_2_O_4_-based lithium-ion batteries.

## Introduction

The applications of lithium-ion batteries (LIBs) consisting of spinel lithium manganese oxide (LMO) are limited at elevated temperatures owing to the severe Mn dissolution [1, 2]. The Mn dissolution from the positive electrode is minimal for the capacity decay of the positive electrode itself; however, the deposition of dissolved transition metal ions on the negative electrode surface degrades the initially formed solid electrolyte interphase (SEI), and a new SEI is formed from further decomposition of the electrolyte on the exposed negative electrode surface [1–5]. This phenomenon consumes the usable Li-ions and the electrolyte solvents in the assembled cell; hence, the cell resistance and capacity fade at elevated temperature cycling and storage with LMO electrodes are very severe. In the past, the dissolution of Mn-ion from LMO is quite serious from Jahn-Teller distortion of the host structure by the intrinsic oxidation number of Mn-ion. Thus, the dissolution of Mn(II)-ion from the disproportionation reaction of Mn(III) in LMO host structure is crucial for the failure of LMO electrode-comprised cells [2, 6, 7]. To improve prompt dissolution of Mn-ion from host structure, the multivalent metal-ion doping of LMO is conducted to increase the average oxidation number of Mn; and hence the dissolution of Mn from Jahn–Teller distortion is significantly suppressed in advanced LMO active materials [8–10]. Nevertheless, the hydrogen fluoride (HF) attack from electrolyte decomposition readily dissolves Mn-ions at elevated temperatures [9, 11] and this failure mechanism is a major concern in advanced LMO chemistry using LIBs. Since the HF formation in the electrolyte solution is readily occurred from the thermal decomposition of LiPF_6_ salt, the suppression of HF attack of LMO electrode is decisive factor for enhancing electrochemical performance of LMO-comprised cells [1, 12, 13]. To enhance the failure of LMO-comprised cells at elevated temperature, highly passivating surface film formation is conducted to reduce the dissolution of Mn from LMO electrode [9, 14–16]. Numerous studies have been reported that the passivation film is formed on the positive electrode surface after initial formation process, and the deposited film significantly affects the electrochemical properties of positive electrode material [10, 17, 18]. Aforementioned deposited organic/inorganic composite film on a positive electrode surface [19–21] suppresses further anodic decomposition of electrolyte components, thereby reducing the transition metal dissolution [22, 23]. However, to the best of our knowledge, the passivation ability of HF or large-sized molecules on the surface film has not been studied, while the attack of HF greatly influences the electrochemical performances of LMO-comprised cells. Herein, a redox indicator was used to evaluate the passivation properties of ethylene carbonate-derived surface films. Whereas the redox-indicator based interpretation of passivation ability of SEI deposited on the carbon electrode is conducted, the physicochemical property of surface film on positive electrode is not well-analyzed [24, 25]. The redox indicator used herein must satisfy two electrochemical and physicochemical properties. One is that the oxidation stability exceeds 4.2 V (*vs*. Li/Li^+^), indicating the redox signal of the indicator. The second is the that the redox potential must be far below the working voltage of the positive electrode. Ferrocene (Fc) is a suitable redox indicator satisfying these two properties [26]. Fc is very stable in carbonate electrolytes with 4.2 V (*vs*. Li/Li^+^) and the redox potential of Fc is near 3.2–3.4 V (*vs*. Li/Li^+^), which is below the working voltage of LMO. Furthermore, the molecular size of Fc (approximately 3.31 Å) [27] is larger than that of the HF molecule (bonding length = 0.91 Å). Thus, the penetration signal of Fc to the LMO electrode surface implies that the HF can attack the LMO surface; hence, the surface film is not sufficient to suppress HF attack from the electrolyte. In this study, the evaluation of the surface film generated from a typical carbonate electrolyte, ethylene carbonate:diethyl carbonate (1.0 *M* LiPF_6_ EC/DEC, 1:1 = *v*/*v*), is sufficient for suppressing the HF penetration. If this proposed method is valid, the expansion of redox-indicator-based experiments for the evaluation of surface films on positive electrode surfaces can be possible.

## Methods/experimental

### Cyclic voltammetry tests

A two-electrode 2032 coin cell was assembled for voltammetry experiments using a LiMn_2_O_4_ (LMO)-coated Al current collector working electrode [90:5:5 *wt*.%; active material:Super P:PVdF polymeric binder (Kureha)], and a polypropylene-polyethylene-polypropylene (PP-PE-PP) separator/Li metal. The 0.1 *M* of ferrocene (Sigma Aldrich, 98%) was dissolved to 1.0 *M* lithium hexafluorophosphate (LiPF_6_) in ethylene carbonate:diethyl carbonate (EC:DEC = 1:1, *v*/*v*) electrolyte and applied to cyclic voltammetry (CV) tests. After assembling the coin cell in an Ar-filled glove box, the coin cell was transferred out for electrochemical characterization.

### Four-electrode beaker cell fabrication

To measure the leakage current on the LMO electrode surface after formation, two working electrode-added four electrode cell was designed. The cell was composed of Li metal/Pt mesh (Sigma Aldrich)/LMO electrode/Li metal. The PP-PE-PP separator was added in each contact of electrodes. For the initial film formation on LMO electrode, 15 mL of 1.0 *M* LiPF_6_ in EC/DEC (1:1, *v*/*v*) electrolyte was added. The 0.2 *C* constant current-constant voltage (0.05 *C* current cut-off) step was applied for five formation cycles. After formation, Fc was added to 1.0 *M* LiPF_6_ in EC/DEC (1:1 = *v*/*v*) electrolyte (0.1 *M*). After adding Fc, the 3.0 V (*vs*. Li/Li^+^) constant voltage step was applied to the Pt mesh electrode to collect the shuttling current of the Fc redox couple.

## Results and discussion

**Fig. 1:**
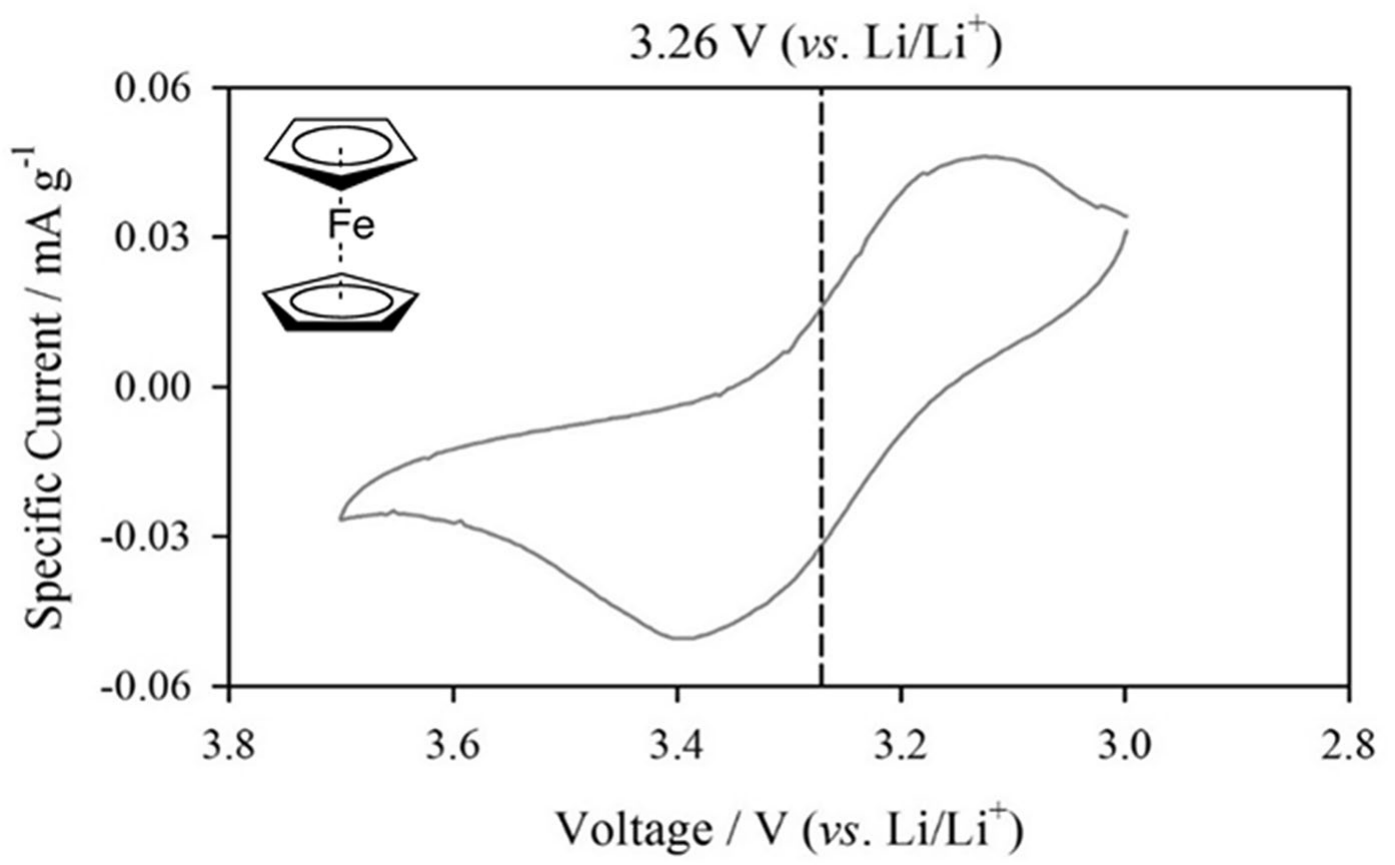
Cyclic voltammogram obtained from 0.1 *M* Fc dissolved 1.0 *M* LiPF_6_ in EC/DEC (1:1 = *v*/*v*) electrolyte at Li/LMO cell with scan rate of 10 mV s^-1^

Figure 1 shows the cyclic voltammogram of Fc on the bare LMO electrode surface. The prerequisites of the redox indicator are summarized as follows: first, the chemical reversibility of the redox couple in carbonate-based electrolyte; this is because the oxidized redox couple migrates to the counter mesh electrode, thereby demonstrating the redox current. Second is the redox potential of the redox indicator. The redox indicator should easily be oxidized on the LMO surface (approximately 4.0 V *vs*. Li/Li^+^) and reduced on the current-collecting electrode surface (3.0 V *vs*. Li/Li^+^). The prerequisites were evaluated by CV. Fc is known to be highly reversible in non-aqueous media, and thus, it is evaluated as a redox indicator for the LMO electrode. The electrochemical characteristics of Fc were measured to evaluate its reversible characteristics in carbonate electrolytes and its redox potential on the LMO surface. In carbonate electrolytes, the reversible characteristics of Fc can be measured by dividing the peak current ratio of oxidation and reduction. Since the oxidation/reduction ratio is close to unity, the reversible characteristic of Fc is maintained in the carbonate electrolyte. Furthermore, the observed redox potential of Fc is 3.26 V (*vs*. Li/Li^+^), which is far below the working voltage of LMO. Since spontaneous charge transfer occurs from the redox potential difference between the LMO electrode and the film-penetrating Fc molecules, Fc can be used as a redox indicator for surface film penetration. Moreover, Fc has a much larger molecular size than HF; therefore, the penetration characteristic of the HF molecule into the formed surface film can be successfully verified.

**Fig. 2:**
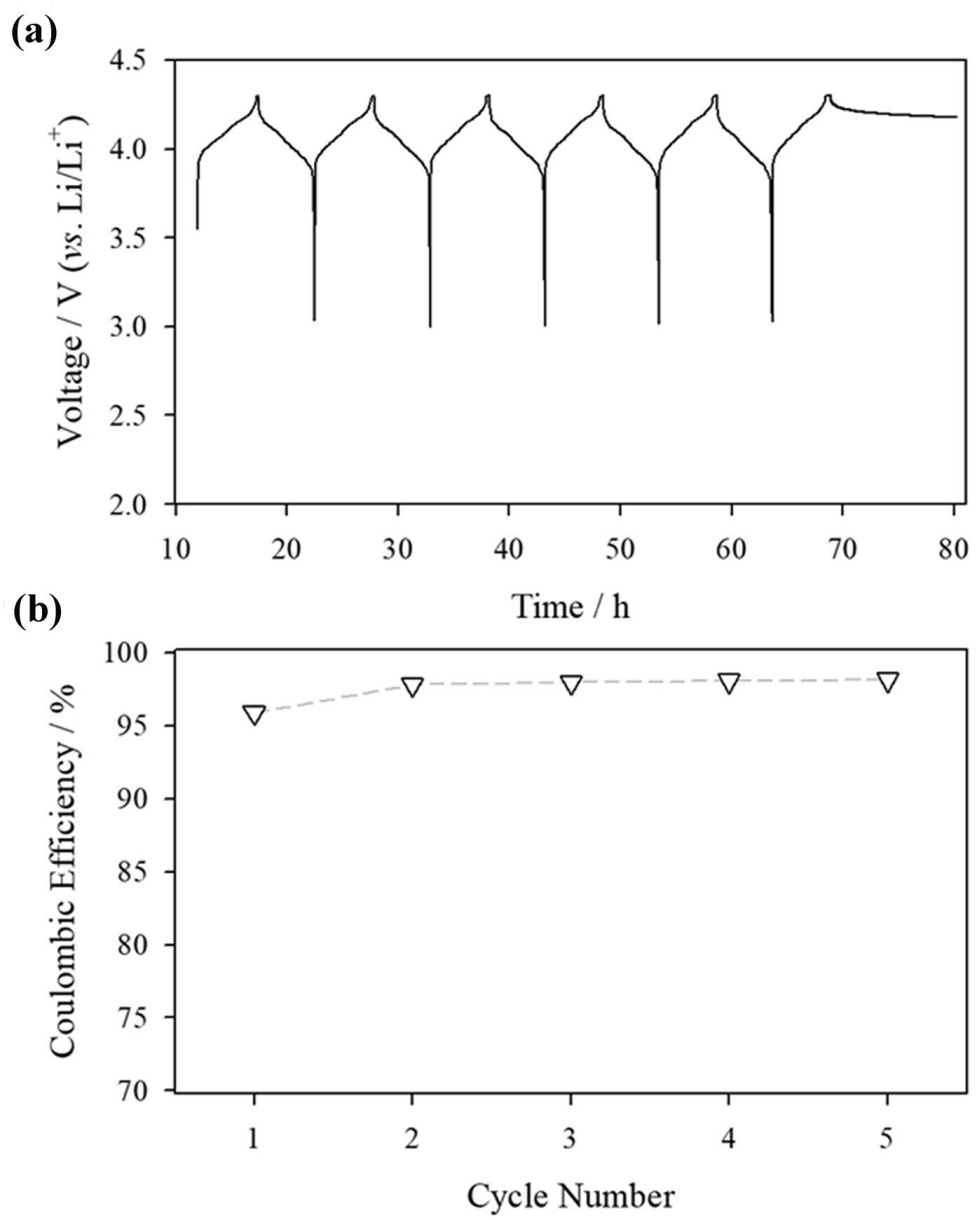
**a** Chronopotentiogram from the 0.2 *C* galvanostatic charge–discharge at the voltage range of 3.0-4.3 V (*vs*. Li/Li^+^) with Li/LMO cell and **b** Coulombic efficiency from the galvanostatic cycling

Before adding the redox indicator, a film was deposited on the pristine LMO surface. Pre-cycling is known as an effective passivating film-forming procedure on the active material surface; thus, pre-cycling was conducted before adding the redox indicator into a four-electrode cell [ref]. Figure 2 shows the pre-cycling profile and Coulombic efficiency of the LMO electrode at room temperature. Because uniform film formation is crucial for the passivation of redox-active molecules, low *C*-rate repeated cycling was conducted. The pre-cycling demonstrated stable time *vs*. voltage curves from the LMO active material. Moreover, the open-circuit voltage (OCV) after the 6th de-lithiation is well retained after a rest period of 10 h. Hence, the film is well-formed on the LMO surface, and the self-discharge of LMO is suppressed from the as-formed surface film. Note that even lithiation and de-lithiation of active material are conducted with two-phase reactions, the severe self-discharge of active material greatly affects OCV values [28, 29]. While the Coulombic efficiency was approximately 95% in the initial cycle, a gradual increase in Coulombic efficiency was observed after repeated cycling and was maintained at over 99 %; hence, the electrolyte decomposition on the LMO surface is focused on the initial formation cycling. From the time *vs*. voltage curve and Coulombic efficiency, effective formation of surface film was obtained.

**Fig. 3:**
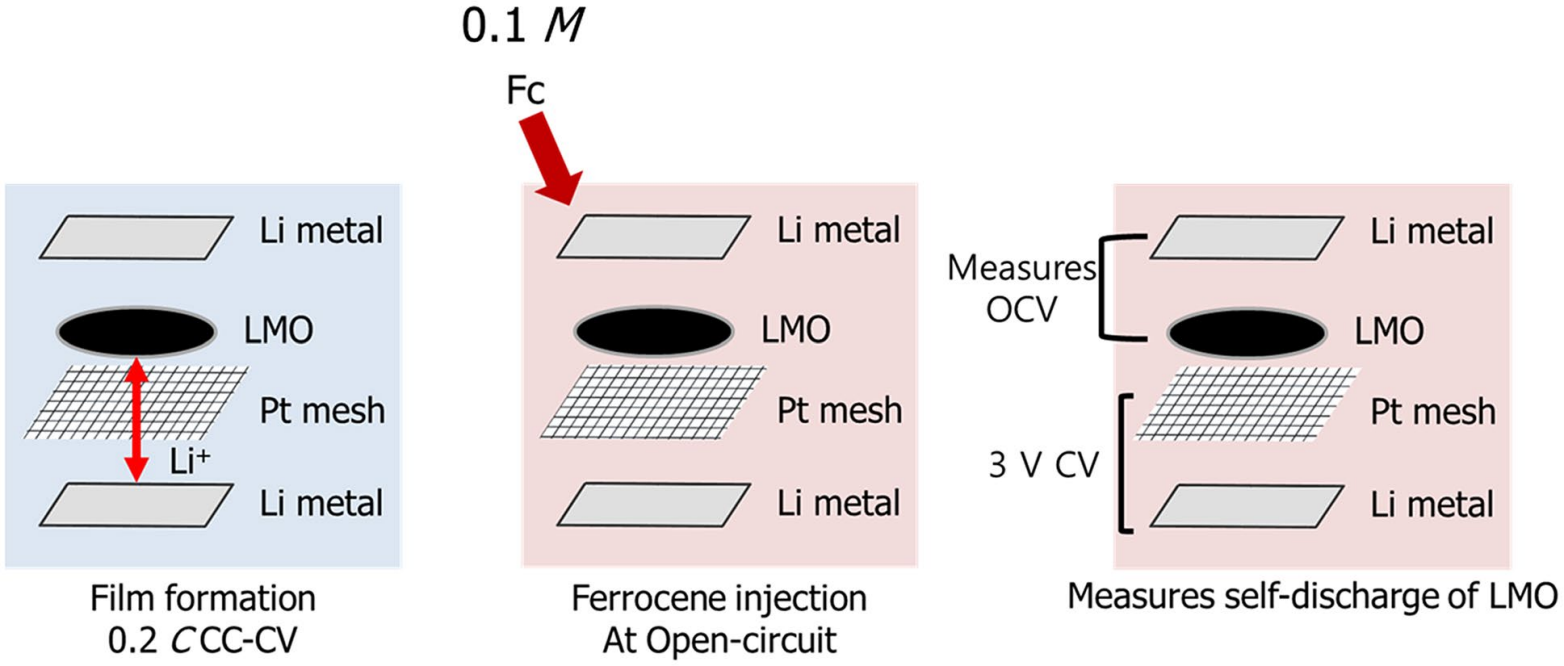
Experimental Scheme for measuring permeability of surface film deposited on positive electrode surface by redox indicator

Figure 3 shows the experimental scheme of the penetrated Fc redox indicator electrochemical measurement. The LMO electrode was pre-cycled five times for the surface film formation. The Coulombic efficiency and OCV retention obtained after fully charging the LMO indicate that the film was effectively formed on the LMO surface, thereby suppressing the self-discharge of LMO. After stabilization of the LMO electrode, Fc was injected into the electrolyte solution at the fully charged LMO electrode comprising the cell. Since the redox indicator is readily oxidized on the LMO electrode surface and diffuses the current-collecting 3.0 V (*vs*. Li/Li^+^) polarized Pt mesh surface, the oxidized Fc is re-collected on the Pt mesh surface. Thus, the self-discharge of LMO electrode from the penetrated Fc molecule was directly observed from the redox current generation. The Fc-induced redox current was further verified by conducting OCV measurements of the LMO electrode. Because the self-discharge of LMO is due to the penetration of Fc into the deposited surface film, lithiation (self-discharge) of the LMO electrode occurs simultaneously. In short, the self-discharge from the Fc penetration is measured by the self-discharge of the LMO electrode and the redox current generated from the ferrocenium (Fc^+^) formation on the LMO surface.

**Fig. 4:**
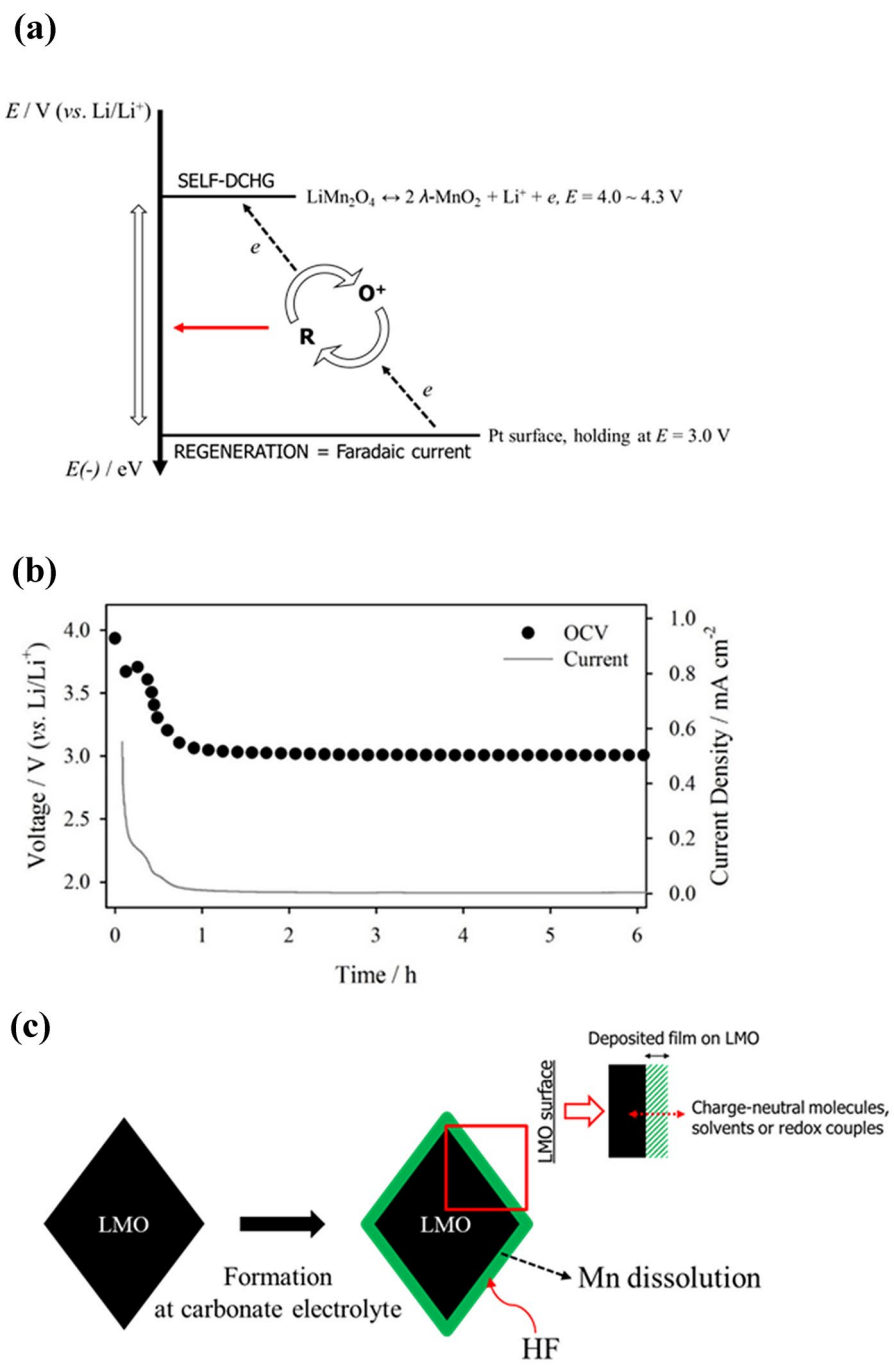
**a** Potential diagram of electrochemical response. **b** Time *vs*. OCV plot from Li/Pt/LMO/Li four electrode cell and time *vs*. current density recorded at 3.0 V (*vs*. Li/Li^+^) polarized Pt mesh electrode. **c** Schematic diagram of the results observed by redox-indicator analysis

Figure 4 shows the OCV values with the oxidation current from the Fc^+^ on the Pt mesh and the analyzed results. Figure 4a shows a summarized diagram of the electrochemical reaction occurring in the four-electrode cell. The four-electrode cell possesses advantage in the individual control and measurement of electrochemical reaction of LMO and Pt mesh electrodes. From the simultaneous measurement of diffusion current from the generated Fc^+^ on LMO surface at Pt electrode and OCV drop of LMO electrode, the precise analysis of self-discharge reaction of LMO electrode can be determined; because the OCV drop only indicates the self-discharge of LMO electrode, which is not identical meaning with generation of Fc^+^ species. The redox potential of Fc is in the middle of the working voltage of LMO and the applied voltage of the Pt mesh electrode; hence, the redox couple is oxidized on the LMO near the surface and regenerated at the Pt mesh surface. Thus, the self-discharge of LMO electrode originates from the charge transfer reaction that occurs on the LMO surface, and regeneration of Fc^+^ to Fc occurs at the Pt mesh surface. This redox shuttle behavior defines the penetration of Fc on the deposited film on the LMO surface. Figure 4b shows the time *vs*. OCV curve of LMO and the collected Fc^+^ current plot. The time *vs*. OCV_LMO_ plot demonstrates the facile self-discharge of the LMO electrode within 1 h of Fc exposure; hence, the possibility of Fc penetration into the surface film on the LMO active material is described. Because the OCV_LMO_ returns to the initial discharged OCV values, the complete self-discharge of the LMO active material is predicted. To define LMO self-discharge from Fc^+^ generation by a charge transfer reaction on the LMO surface to Fc, the diffusion current from the as-generated Fc^+^ is recorded on the Pt mesh surface. The self-discharge of LMO was observed from the penetration of Fc into the LMO surface, since the potential of Pt electrode was 3.0 V (*vs*. Li/Li^+^), the reduction of Fc^+^ is readily conducted on the Pt surface because of the high overpotential. The chronoamperogram shows the Cottrell-like current behavior at the Pt mesh surface; hence, the diffusion of Fc^+^ is explained. Furthermore, the diffusion current syncs with the OCV decay profile; thus, the poor passivation ability of the surface film deposited on the LMO electrode is the main cause of the self-discharge. A schematic of the carbonate-derived surface film is shown in Fig. 4c. During the initial pre-cycling, the LMO surface was passivated from the carbonate-decomposed products. While the surface film deposited on the LMO surface further suppresses the electrolyte decomposition on the LMO surface, the penetration of charge-neutral molecules cannot be prevented. Hence, the self-discharge of the LMO electrode readily occurs due to the charge transfer reaction to the redox indicator molecule. The molecule used in this study had ideal size approximately 3.31 Å [27]; therefore, the penetration of HF molecules into the surface film is readily possible in conventional carbonate-derived films. In conclusion, the blocking ability of the HF molecule in the deposited surface film is crucial for the enhancement of LMO-comprised LIBs.

## Conclusions

The Fc molecule is introduced as the redox indicator for the surface film passivation ability of the LiMn_2_O_4_ (LMO) positive electrode. From the charge transfer of LMO to the penetrated Fc molecule near the LMO surface, the self-discharge of the LMO electrode and the diffusion current from ferrocenium molecule is observed. The surface film on LMO derived from the carbonate electrolyte suppresses continuous electrolyte decomposition, while the infiltration of Fc molecules is not obstructed. Because the Fc molecule is larger than the LMO-attacking HF molecule, the degradation of LMO surface from the HF attack cannot be restrained in conventional carbonate-derived films. Therefore, to improve the LMO-comprised LIBs, a positive electrode surface passivating functional electrolyte is needed to prevent HF from approaching the LMO surface. Hence the surface film modification of LMO to suppress the penetration of HF is crucial for enhancing the electrochemical performance of LMO electrode.

## Data Availability

The datasets used and/or analyzed during the current study are available from the corresponding author upon reasonable request.

## Abbreviations

LMO: Lithium manganese oxide
HF: Hydrofluoric
LIBs: Lithium-ion batteries
Mn: Manganese
SEI: Solid electrolyte interphase
Fc: Ferrocene
EC: Ethylene carbonate
DEC: Diethyl carbonate
PVdF: Polyvinylidene fluoride
Pt: Platinum
PP: Polypropylene
PE: Polyethylene
CV: Cyclic voltammetry
OCV: Open-circuit voltage
